# Letter from Augusta

**Published:** 1885-11

**Authors:** Wm. H. Doughty

**Affiliations:** Augusta, Ga.


### LETTER FROM AUGUSTA.

### THE LOCAL OPTION BILL AND THE MEDICAL PROFESSION.

*To the Profession of Georgia:*

Temperance legislation has culminated in the passage by the Legislature of a "Local Option Bill" for the State, the final enforcement of which, however, depends upon the popular vote in counties not now classed as prohibition counties. Before this is done, it is well for the profession to consider how the bill affects them and the sick whom they represent. In general terms, it prohibits the sale of all spirituous and fermented liquors, except domestic wines and cider, and prevents druggists from dispensing, for medicinal purposes, any form of alcoholic stimulants, other than "pure alcohol." Now, then, it does the profession the violence of severing their relations with the druggist, through whom all supplies naturally come, having first placed it out of their power to obtain alcoholic stimulants, for dispensing in person to their patients, by forbidding their sale elsewhere. No resource is left for obtaining these valuable medicines, except by importation or surreptitiously.

Shrewdly enough the bill does not prevent the physician from prescribing alcoholic stimulants in any form, but denies to him and to his patient the possibility of legally getting anything but "pure alcohol." Manifestly the object was to prevent the use of alcoholic stimulants as medicine, as far as possible, by law, thereby placing us also in ignominious classification with those who sold them as a beverage. One effect, therefore, of this legislation is to obstruct, yea, virtually nullify, the exercise of vested rights obtained by the physician in his diploma.

We repeat, the purpose in this bill was unmistakably to reach the prescriber and the thing prescribed also—the physician as well as the pharmacist and dealer in liquors. The character of the debate, while the bill was on its passage, leaves no room for doubt on this point, for, after the Senate had adopted an amendment allowing druggists to fill physicians' prescriptions for alcoholic stimulants, it was promptly reconsidered and rescinded, the convincing statement bringing it about, being the abuse of the privilege by both parties, especially the physicians. The animus of this legislation renders it probable that if it had been within the power of the Georgia Legislature to deny to physicians the right to *prescribe* alcoholic stimulants, as they had denied druggists the right to dispense them, it would have been done. Having been guilty of the folly of enacting "pure alcohol," a full and sufficient substitute for them, there is no good reason why they might not have done this also. As it is, they gave us the shadow instead of the substance, having left the right to prescribe, but denied the opportunity of exercising it.

Moreover, the Local Option Bill, in effect, libels the good citizenship of the profession by insinuating a want of integrity in the use of alcoholic stimulants as medicine, and by certifying a collusion with druggists for an evasion and violation of law. This distrust conveys dishonor, and along with the rape of the materia medica perpetrated by expunging all forms of alcoholic stimulants, other than "pure alcohol," warrants the just indignation of a profession whose natural ties are in sympathy with the correction of all physical and moral abuses. In the opinion of the writer, the occasion demands an outspoken protest, as well for the defense of the rights, interests and honor of the profession as to rebuke the presumptuous legislation that decides questions in medicine raised by politicians and social reformers of every kind. Its voice should be heard in its own behalf, and in dignified remonstrance against the iniquities of this bill. Denied a hearing before the court, convicted by the *ex parte* testimony of men whose hope of political promotion largely depended upon their zeal in the temperance cause, shall we plead guilty to the indictment by unmurmuring acquiescence in the act? Or, ere the sentence and the stigma be affirmed by the highest tribunal, the electors at the polls, seek in self-defense so to enlighten the public mind as possibly to defeat the measure? The repository of the trust and confidence of our fellow-citizens; holding in our possession and keeping the very secrets of their personal and domestic lives, I cannot believe they will willingly affirm that the profession, as a body, are unworthy to be trusted in this particular. Demagogues have no scruples; sentimentalists "know not what they do." The only sentiment that governs the profession in its relations to alcohol and its use as a medicine is *duty*. With the political and politico-religious aspects of the Local Option Bill we have no concern. While many of us are in close sympathy with the effort to reform the abuse of alcohol as a beverage, we claim the right to withhold or to prescribe it as the interests of the sick may demand.

So much prominence has been given of late to the discussion of alcohol as a beverage that its importance as a medicine has been lost sight of to a degree. Its high rank and distinctive value have been compromised by the artful teaching that its medical application simply gilds it as a beverage.

Before the public we can formulate the position of the profession in the present issue in this wise:

1st. Alcohol concerns the profession only as a medicine; as such it takes high rank with opium and quinine.

2d. It is indispensable in the treatment of many diseases, and that no substitute has been found for it.

3d. That alcohol proper is "never given internally" (Wood); does not represent all the qualities of the different forms of alcoholic stimulants which alone are used internally; and cannot be made to meet the varying emergencies of disease that determine the selection of the particular one necessary.

4th. That alcoholic liquors (distilled and fermented) differ in their respective physical, chemical and therapeutical properties, which distinctive differences determine their selection in appropriate cases. Deriving their properties as *stimulants* from varying proportions of alcohol, they nevertheless possess other valuable properties that cannot be ignored, and for which they are employed.

5th. The condition and interests of the sick determine the time and occasion of their use respectively, the physician alone being the proper judge. A choice of remedies must be awarded the sick under all circumstances, a maxim which no plea of morality or affectation of interest can set aside.

6th. To legalize the practice of medicine, and then deny, directly or indirectly, to the practitioner and the sick the use of necessary medicines involves a breach of public faith, a mockery of science as well as of human philanthropy.

7th. That "pure alcohol," as authorized to be dispensed by druggists, in the Local Option Bill, can better serve the purposes of a beverage than a medicine. The old toper may water and sweeten it to his taste, while to the sick it would prove repugnant, if not injurious, or at least unsuited.

It is in evidence, however, that in counties where local option now prevails, some physicians have abused their privilege by prescribing stimulants, knowing that they were to be used as a beverage. This is not denied, but does it justify libeling the whole body of conscientious men who make up the profession at large? It would be more remarkable if a few unscrupulous members were not found in so numerous a body. Our legislators seem to forget that the annals of legislation are not free from instances of corruption ; that the lobby stands hard-by the halls of legislation, where members are sometimes unduly influenced in the passage of public measures ; that it is said free railroad passes are accepted by some, even of the very legislators whose pleasure it is to libel as untrustworthy the physicians of the State. Because this is so, shall we assume that all legislators are corrupt ? Nay, rather let us invoke for them the justice we claim for ourselves, and make sufficient answer to the respective charges by saying, punish the guilty alone by appropriate legislation.

But morality has been plead in extenuation of the bill, even in its restrictive features. Well, as against the evils of intemperance, as involved in the sale and use of alcoholic stimulants as a beverage, no objection is made to it. Even the best moralists may differ as to the wisdom of prohibition, but the moral plea becomes an instrument of torture when turned against the sick, helpless and infirm. The physician here presses himself into notice in a dual capacity ; he cannot divest himself of an identity with the interests of the sick whom he likewise represents. Where is the morality or Christian philanthropy that withholds from the sick what he most needs when life is ebbing away or strength failing ? Make it a penal offense to fill a prescription for wines or other spirituous liquors than "pure alcohol," knowing that the latter cannot be made a uniform, universal, acceptable substitute, and that moral pretext becomes a mere subterfuge. A pulpit infected with the contagion of politics, or a legislature pursuing a phantom of excellence beyond human attainment, may act thus, but the welfare of the sick is the shibboleth of the profession, from which they can neither be seduced nor driven. It is the province of the physician to advise or prescribe alcoholic stimulants when needed. He cannot and does not dispense them, so that the responsibility for withholding from the sick and suffering what is needed must rest with the proper parties. It is well, therefore, for the public, before voting this bill into operation, to know that physicians cannot keep a stock of liquors as medicines, so that if they, at the bidding of the Legislature, vote them beyond reach, they must suffer the consequences. Does anyone believe that a sane person would vote a necessary article entirely beyond his reach ? For this reason, it is believed that timely enlightenment of the public may check the progress of prohibition carried to this extreme ; may lead them to retain as a medicine, when sickness invades their households, what is decried as a beverage. Or, if prohibition in this form is not voted down, may possibly predispose them to an early repeal of the clause prohibiting the dispensing of alcoholic stimulants as a medicine by those who dispense other medicines. To this end, let the profession, first remonstrating against such legislation, as not merely idle and foolish, but unjust and injurious, do all in their power to enlighten the public as to their true interests.

The Medical Association of Georgia does not meet until April next, by which time the people will have acquiesced in the *unquestioned* attitude of the bill toward the profession, and like our legislators, in their ignorance or folly, possibly have voted its adoption. Let every physician, each in his own way, within the limits of his own influence, exert himself for the desired result.

Respectfully,

WM. H. DOUGHTY, M. D.

Augusta, Ga., October, 1885.

